# The A B C of Vitamins

**Published:** 1922-02

**Authors:** Harold Scurfield

**Affiliations:** (formerly Medical Officer of Health, Sheffield)


### THE A.B.C. OF VITAMINS.

By HAROLD SCURFIELD, M.D., D.P.H. (formerly Medical Officer of Health, Sheffield).

THE new knowledge about vitamins has aroused widespread interest. Its effect on the community varies with the individual temperament. For some of us who "spend our time in nothing else but either to tell or hear some new thing," knowledge does not advance quickly enough. Some are already bored at the mention of the word "vitamins." Certain people have conceived the idea that foods which do not contain vitamins are of no use. Some regard the matter as so complicated that they give it up as a bad job. Others rush after each proprietary article which comes out guaranteed by advertisement to contain all the vitamins. It may be useful, therefore, to sum up the position and endeavour to show that the present knowledge can be utilised greatly to the benefit of the community without any scientific study and without adding to the complications of the problem of leading a healthy life.

It is clear that vitamins are necessary for the normal growth and development of the young. An insufficiency of A may lead to rickets, defective teeth, a form of eye disease, and lowered resistance to the onslaught of various diseases. An insufficiency of B may cause beri-beri, and of C may result in the development of scurvy. Speaking generally, when outbreaks of any of the above diseases occur there are always numbers of persons suffering from slight or ill-defined symptoms, the origin of which would not be suspected if it were not for the presence of the well-defined cases. It is well, therefore, that we should play for safety and endeavour to secure a safe diet for all. Let us look at the matter in another way. If we could abolish rickets, defective teeth and scurvy by careful attention to dietary, the improvement in the public health would be astounding.

Perhaps at the outset we can lay down a few comforting propositions. In the first place, the new knowledge confirms beliefs already strongly held as to the value of certain foods, *e.g.*, of milk, oranges, green vegetables, eggs, cod-liver oil and wholemeal bread.

In the second place, the cooking problem is rendered simpler by the enhanced value given to raw, green vegetables such as lettuce, watercress, to tomatoes, and to raw fruit such as oranges and bananas. Vitamins, in fact, favour the simple life.

In the third place, although we have no accurate knowledge as to the exact amount of each vitamin contained in a given food, we can rest assured that there is no difficulty in choosing a dietary which contains a sufficiency of each without having recourse to patent foods. Professor Mellanby considers that if we each had an egg, an orange and a pint of milk daily, it would not matter much what else we ate. Dr. Hamill (in the pamphlet recently issued by the Ministry of Health) expresses the opinion that the addition of milk and green vegetables to most ordinary dietaries would make them safe.

In the fourth place, the discovery of vitamins does not involve the scrapping of rules with regard to the proportion of protein, fat, starch, sugar, salts and water, and the calculation of diets based on calories, but it does warn us that the choice of the kind of protein or fat is important, and that in fact quality is important as well as quantity.

In the fifth place, though many of the vitamin-containing foods are expensive, the choice of a satisfactory diet is doubtless within the reach of most of us if care is taken to economise on useless articles such as alcohol and sweets.

Let us consider for a moment the practicability of the Mellanby rule of an orange, an egg and a pint of milk per day, with the addition of the green vegetables recommended in the Ministry's pamphlet.

Except from August to October, oranges could be bought for about a penny each during 1921. During the dear orange season good tomatoes for eating raw could be bought at 4d. or 5d. a pound, and raw tomatoes, as a source of the antiscorbutic vitamin, are an excellent substitute for oranges. Tomatoes become more plentiful year by year, and the dear season for oranges will gradually disappear, as South African oranges become more plentiful. New-laid eggs were expensive most of last year, but fortunately dried eggs, which are usually about one-half the price of new-laid, are just as valuable sources of the A and B vitamins. Dried eggs can be "scrambled" or "buttered," and make excellent omelettes.

The daily pint of milk is important, not only for its vitamin content, but also as a source of calcium for bones and teeth, and of a protein of high biological value. It has been shown that milk will prevent and cure pellagra, a disease which attacks populations living largely on maize, the protein of maize being of low biological value. Raw milk must not be given to children. I have no hesitation in recommending that the pint of milk takes the form of 20 measures of a reliable brand of dried milk. Milk dried by the hot-roller process retains its A and B vitamins, and if it loses any of the small amount of C which it contained, this does not matter because we look to the orange for our supply of C. It is important that the dried milk should be the produce of cows fed on green food all the year round. A point which is frequently lost sight of is that in drying all the food value of the milk is retained, whereas in the "scalding" of milk in an ordinary household the milk is often much impoverished. Even if the milk is not allowed to boil over, cream is lost with the skin and coagulated lactalbumin sticks to the inside of the pan and goes down the sink.

Green vegetables are useful as a source of both A and C vitamins. They have been expensive lately. Raw greens are the best. Lettuces are very easy to grow on allotments, and watercress could no doubt be produced in much greater quantities in this country if its value were realised. The important point to note about green vegetables like cabbages, spinach, &c., is that they should be cooked for the minimum time. Probably steaming is better than boiling, so that nothing may be lost in the water.

